# Inflammatory Bowel Disease Through the Lens of Single-cell RNA-seq Technologies

**DOI:** 10.1093/ibd/izaa089

**Published:** 2020-05-09

**Authors:** Daniele Corridoni, Thomas Chapman, Agne Antanaviciute, Jack Satsangi, Alison Simmons

**Affiliations:** 1 Medical Research Council (MRC) Human Immunology Unit, MRC Weatherall Institute of Molecular Medicine (WIMM), John Radcliffe Hospital, University of Oxford, Oxford, UK; 2 Translational Gastroenterology Unit, Nuffield Department of Medicine, Experimental Medicine Division, University of Oxford, John Radcliffe Hospital, Oxford, UK; 3 MRC WIMM Centre for Computational Biology, MRC Weatherall Institute of Molecular Medicine, John Radcliffe Hospital, University of Oxford, Oxford, UK

**Keywords:** mucosal immunology, inflammatory bowel disease, single-cell RNA-seq, CITE-seq, CyTOF, personalized medicine

## Abstract

The intestinal mucosa represents a unique environment where the coordinated function of diverse epithelial, mesenchymal, and immune cells maintains a physiologically balanced environment in the presence of gut microbiota. The intestinal mucosa plays a central role in the pathogenesis of inflammatory bowel disease (IBD), yet the molecular and cellular composition of this diverse environment is poorly understood. However, the recent advent of multimodal single-cell technologies, including single-cell RNA sequencing (scRNA-seq), now provides an opportunity to accurately map the tissue architecture, characterize rare cell types that were previously overlooked, and define function at a single-cell level. In this review, we summarize key advances in single-cell technology and provide an overview of important aspects of computational analysis. We describe emerging data in the field of IBD and discuss how the characterization of novel intestinal mucosa cell populations is reshaping our understanding of this complex disease. We conclude by considering the potential clinical applications, including the definition of novel drug targets and the opportunity for personalization of care in this exciting new era of precision medicine.

## Teaser

This review describes the recent advent of multimodal single-cell technologies, including single-cell RNA sequencing, and discusses how characterization of novel intestinal mucosa cell populations in health and IBD may have potential clinical applications, including drug discovery and personalization of care.

## INTRODUCTION

The gastrointestinal mucosa is the largest immunological organ in the body and represents a uniquely challenging environment, where a careful balance must be maintained between tolerance and immune response in the presence of a huge bacterial burden. When this balance fails, intestinal inflammation may develop, as observed in inflammatory bowel disease (IBD).^1^

The molecular characteristics of mucosal cells driving the pathology of IBD are poorly mapped, primarily because rare intestinal cells have previously been challenging to isolate and characterize in a nonbiased fashion. Classical approaches, such as bulk-RNAseq experiments, are limited to the collection of specific cell types or tissues, with the belief that these populations of cells are homogenous. However, it is now understood that these approaches can only provide the average expression of genes deriving from similar, but not identical, cell types that may be in quite different developmental stages. In contrast, single-cell RNA-seq (scRNA-seq) provides the full transcriptome at a single-cell level, allowing high-resolution views of cellular heterogeneity within specific and well characterized cell populations. Single-cell technologies, such as scRNA-seq, are now enabling granular analysis of the cell types that make up human tissues, and revealing the nature of their remodeling during disease.^2, 3^ The unprecedented detail that these technologies provide is leading to the identification of profound cellular heterogeneity in each component of the intestinal mucosa. Using computational analysis, scRNA-seq allows grouping of cells according to their gene expression states, characterization of transitions, and reconstruction of lineage decisions and intercellular gene networks.^4^

Since the first description of scRNA-seq in 2009, single-cell technology has led to significant advances both in the discovery of novel cell types and their breakdown in diverse diseases, including IBD.^5–8^ Indeed, single-cell technology now promises a potential revolution in our understanding of IBD pathogenesis and may drive a new era of drug discovery and personalization of care. In this review, we will summarize key advances in single-cell technology and discuss how recent discoveries, including the characterization of novel cells in the intestinal mucosa, are reshaping our understanding of IBD.

## SINGLE-CELL RNA SEQUENCING TECHNOLOGY AND WORKFLOW

Following the first scRNA-seq approach reported by Tang and colleagues,^5^ multiple methods for single-cell transcriptomics have been developed over the last decade. The key differences between these technologies lie in how and which RNA transcripts are amplified and how cell-of-origin information is preserved, forming the basis of trade-offs between throughput, sensitivity, and cost per cell.

Some methods generate full-length cDNA libraries from RNA, enabling better characterization of isoforms and longer transcripts, whereas others retain only the 3’ or 5’ end of the transcripts.^9–11^ Typically in both approaches, the analytes are limited to eukaryotic mRNAs due to polyT priming; although recently, total RNA methods have also emerged, enabling capture of non-polyadenylated RNA species.^12^ In contrast to these largely unbiased methods, targeted scRNA-seq platforms allow specific capture and quantification of a predetermined selection of transcripts, decreasing costs and increasing sensitivity, in particular for low abundance transcripts. Commercial availability of immunology-focused panels (eg, BD Rhapsody Single-Cell Analysis System, Franklin Lakes, NJ, USA) make it particularly suited to studying immune populations but currently limiting for other cell types.

**FIGURE 1.**
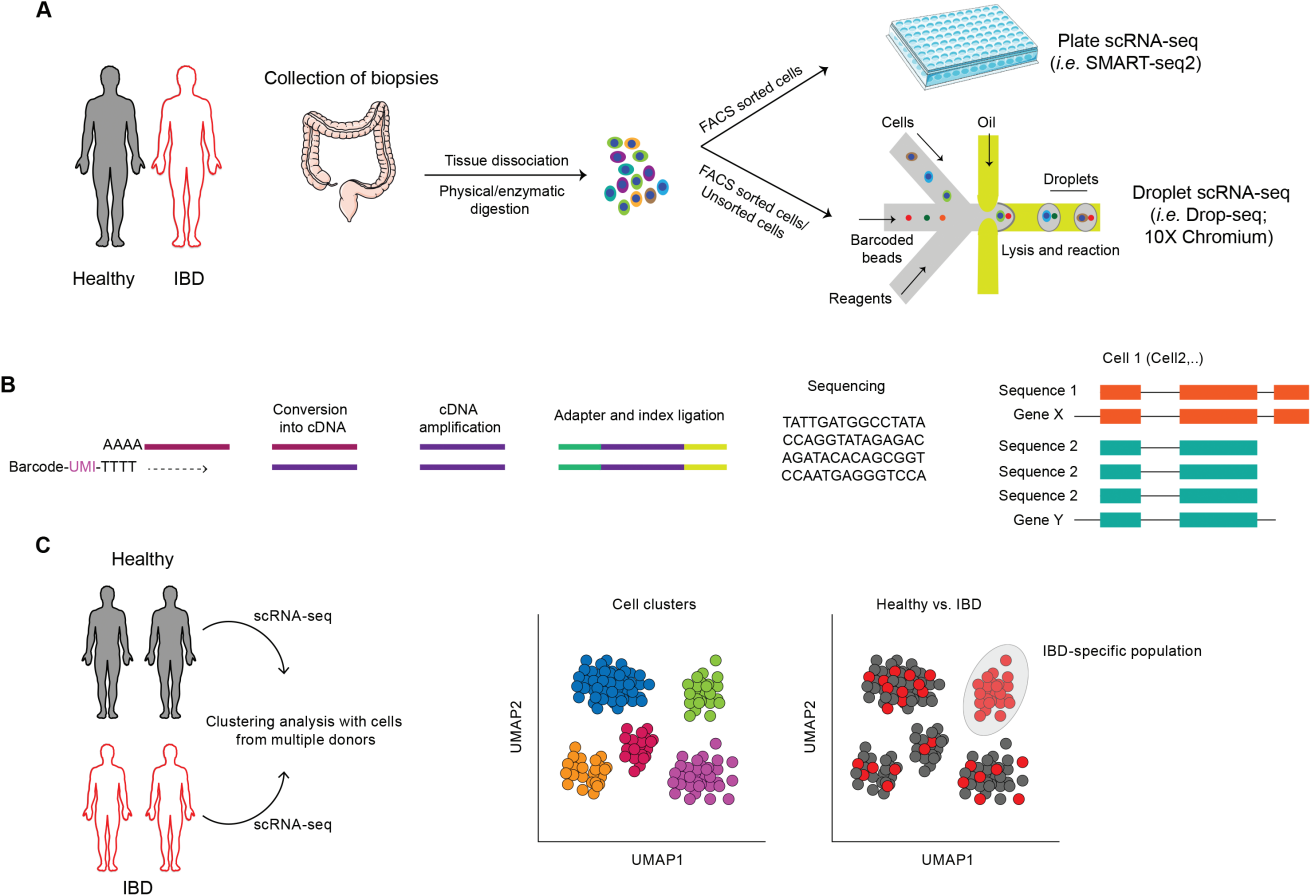
Mapping cells in intestinal mucosa. A, Intestinal biopsies collected from healthy donors and IBD patients are dissociated to obtain suspensions of cells that can be used to further separate specific subsets (ie, FACS sorted cells) before being subjected to scRNA-seq by either plate-based sequencing approach or droplet-based approach. B, RNA derived from single cells is converted to cDNA which is then amplified, adapters are added, and samples pooled and sequenced on high-throughput platforms. C, Computational analysis of scRNA-seq allows unbiased mapping of novel subsets of cells, uncovering rare cell populations and ultimately identifying disease-specific cell subpopulations.

Regardless of the choice of chemistry, in low throughput approaches, single cells are typically sorted into plates, generating and indexing unique sequencing libraries in each (eg, SmartSeq2; Fig. 1A). As such, this is limited by plate size and the available sequencing index combinations, although higher throughput combinatorial-index approaches have also been developed.^13^ In high-throughput methods, on the other hand, transcript cell-of-origin information is recovered by distributing a single-cell suspension across microwells or droplets (eg, 10x Genomics, InDrop), each containing a unique barcode that is incorporated into cDNA during in situ reverse transcription (Fig. 1A). In addition, individual RNA molecules are often labeled with a “unique molecular identifier” (UMI), a randomly generated barcode that is preserved over multiple rounds of polymerase chain reaction (PCR) amplification, allowing it to eliminate the effect of both PCR bias and optical duplicates generated during sequencing^14^ (Fig. 1B).

High-throughput devices enable the analysis of large numbers of cells in a single experiment and are best suited for characterization of highly heterogeneous samples and discovery of rare cell types or states. The main drawbacks, however, include lower RNA capture efficiency and low rate of cell encapsulation, thus requiring larger amounts of starting material.^15^ Furthermore, additional noise and artifacts can often be introduced by capture of cell-free ambient RNA, formation of cell and barcode multiplets, and/or encapsulation of cytoplasm-free nuclei. Thus, for the analysis of largely homogenous samples, low-throughput plate-based assays can prove superior, providing increased sensitivity for detection of low abundance transcripts.

A typical single-cell transcriptomics experiment generates millions of single- or paired-end short sequence reads. These are first demultiplexed and undergo a number of quality control steps: trimming of poor quality bases and nonbiological adapter and/or primer sequences; sequencing error correction in UMI and/or cell barcode sequences; and identification of potential contaminants. Short reads are then aligned to the reference genome using a splicing-aware aligner such as STAR,^16^ and alignments are summarized as read or UMI counts per cell based on overlaps with reference transcript annotations. Alternatively, raw count matrices can be obtained with alignment-free methods, such as kallisto.^17^

Raw count matrices are then subject to further quality control, including identification of debris and poor quality cells, empty drops or wells,^18^ multiplets^19^ or free nuclei. Further correction of counts for Illumina index-swapping^20^ and imputing dropouts can also be undertaken.^21^ The data are then normalized, scaled, and batch-corrected before selection of highly variable features used as input for dimensionality reduction techniques such as PCA. Supervised or unsupervised clustering can be performed to identify groups of cells with similar transcriptome profiles, which typically correspond to different cell types or states. Clusters can be visualized by further embedding the data into 2 or 3 dimensions using T-distributed Stochastic Neighbor Embedding or Uniform Manifold Approximation and Projection algorithms (Fig. 1C). Differential expression and differential abundance analyses enable the researcher to identify both changes in cell type composition and transcriptomic differences within the same cell types between samples of interest. Computational analyses are discussed in greater depth elsewhere.^22–25^

## COMBINING THE TRANSCRIPTOME AND PROTEOME IN SINGLE-CELL ANALYSIS

The strength of scRNA-seq lies in its sensitivity and the ability to quantify gene expression in an unbiased fashion. Although gene expression analysis is hugely important, it does not describe protein levels or post-translational modification. Combining single-cell analysis of the proteome complements and validates scRNA-seq and enables generation of high-dimensional multimodal single-cell data.

**FIGURE 2.**
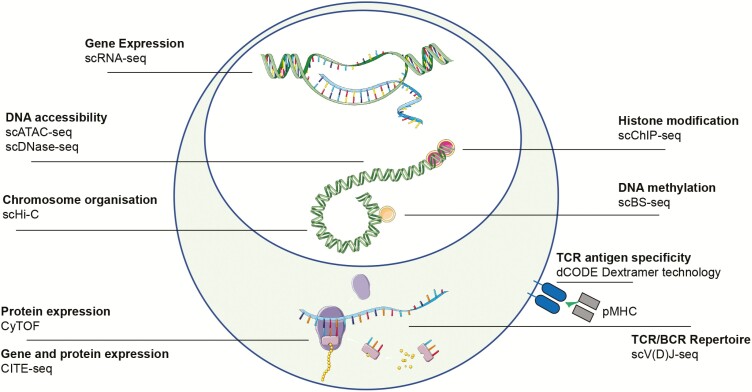
Single-cell techniques. An overview of the methodologies available to interrogate molecular layers at a single-cell level. Abbreviations: single-cell combinatorial indexed Hi-C, sciHi-C; cytometry by time of flight, CyTOF; single-cell chromatin immunoprecipitation, scChIP-seq; single-cell bisulfite sequening, scBS-seq; single-cell T/B Cell Receptor sequencing, scTCR/BCR-seq.

Cytometry by time of flight (CyTOF) has developed alongside scRNA-seq and represents an essential tool for single-cell analysis of the proteome^26^(Fig. 2). Cytometry by time of flight allows simultaneous quantification of over 40 cellular parameters and is a major improvement over multiparameter flow cytometry, although it is significantly more expensive. A variety of algorithms have been developed for computational analysis, which can define cellular proteomic heterogeneity at single-cell level.^27–29^

Cytometry by time of flight was employed to investigate whether the apparent mesenchymal stromal remodeling observed in IBD using scRNA-seq also occurred at protein level. This resulted in an extensive validation of the majority of the transcriptomic signatures, confirming that mass cytometry can capture key parameters of pathogenic colonic mesenchymal behavior in IBD that correlate with clinical disease activity.^7^ A number of algorithms have been developed to integrate multimodal single-cell data sets.^30, 31^ For example, Zhang and colleagues developed a computational strategy based on canonical correlation analysis (CCA) to integrate multimodal transcriptomic and CyTOF profiles at single-cell level as a tool to define the cell populations that drive joint inflammation in rheumatoid arthritis.^32^

Unbiased analysis of the proteome coupled with simultaneous transcriptome analysis at single-cell level has also been recently developed. For example, using REAP-seq technology, cells labeled with antibodies conjugated to DNA barcodes can be used for sequencing.^33, 34^ Another method called Cellular Indexing of Transcriptomes and Epitopes by Sequencing (CITE-seq) combines multiplexed protein marker detection with unbiased scRNA-seq^35^ (Fig. 2). Here, antibodies are conjugated to oligonucleotides, using streptavidin-biotin interaction. Oligonucleotides can be captured by oligo-dT primers, used in scRNA-seq library preparation, that have a barcode for identification that can be amplified by PCR. Both mRNA and antibody-derived oligos are indexed by a shared barcode during reverse transcription. Single-cell suspensions are stained with antibodies after standard flow cytometry protocols and processed for scRNA-seq.

Finally, to enable pooling of multiple samples for loading of commercial scRNA-seq platforms and reduce costs, another approach called “cell hashing” has been developed.^36^ This is based on the principle that a defined set of oligo-tagged antibodies against ubiquitous surface proteins can be used to label different experimental samples. Cells from different samples are stained with DNA-barcoded antibodies, and these distinct barcodes allow pooling of multiple samples into one scRNA-seq experiment. After sequencing, cells are assigned to each sample based on their hashtag-oligos. In addition to reduced costs, multiplexing multiple samples together can greatly increase data quality by allowing identification of cell multiplets and eliminating batch effects that may otherwise confound comparative analyses.

## RECONSTRUCTION OF T CELL RECEPTOR PROFILING REVEALED BY SCRNA-SEQ

During their development in the thymus, T cells acquire the capacity to recognize foreign antigens through the expression of polymorphic T cell receptors (TCRs).^37^ These TCRs are composed of 2 glycoprotein chains; alpha (α) and beta (β) chains represent more than 90% of the total T cell population, while gamma (γ) and delta (δ)chains are a much smaller subset. A highly diverse T cell repertoire is ensured by random recombination of the variable (V), diversity (D), and joining (J) segments in TXPβ chains, the V and J segments in TCRα chains, and also by the addition or deletion of nucleotides at the junctional sites. The resulting sequence coded by the V(D)J junction, known as CDR3, allows a T cell to recognize a specific antigen presented by the major histocompatibility complex (MHC) molecules.^38^ Knowledge of the TCR repertoire is critical to the understanding of the dynamic changes that occur in T cells when responding to antigen in cancer or inflammatory disorders.

ScRNA-seq has revolutionized TCR analysis, which had previously been limited by the “bulk approach,” which poorly defined pairing of the αandβchain, hindering understanding of the function of specific T cells in vivo (Fig. 2). Single-cell sequencing protocols are divided into tag-based or full-length cDNA approaches.^39^ The full-length approach was used to investigate the TCR by Stubbington and colleagues in 2016, who developed the first computational method, called TraCeR, to reconstruct paired TCRα and β chains in cells isolated from mouse spleen using scRNA-seq with SMART-seq2 protocol.^40^ The most recent approaches now take advantage of the commercially available emulsion-based microfluidic platform, the Chromium 10X, which allows analysis of both the TCR repertoire and gene expression profile from the same single cells. This is a powerful approach to gain insight into the lineage, clonal dynamics, and connectedness of diverse T cells.

Single-cell TCR repertoire analysis has recently been used to explore the long-term persistence of resident CD8^+^ T cells in transplanted small intestine.^41^ This study demonstrated that persisting resident CD8^+^ T cells survive over 1 year in transplanted duodenum, and a limited number of expanded clones characterize the TCR repertoire of these cells. Interestingly, lamina propria and intraepithelial resident CD8^+^ T cells present a similar immune repertoire and share a fraction of clones, suggesting that common progenitors can be dynamically primed into phenotypically different subpopulations of intestinal memory CD8s. Similar analysis may be employed to delineate the extent of phenotypic plasticity of T cells in the intestinal mucosa during IBD. Importantly, although in IBD a solitary antigen is unlikely to explain the complexity of T cell evolution in the inflamed intestinal mucosa, tracking clonal expansion of specific subsets of T cells may reflect responses to dysbiotic commensals, which is a hallmark of this disease.^42^

ScRNA-seq of TCR can also be used to guide the development of novel experimental models to study the effect of commensal-specific T-cell responses. In fact, as demonstrated by Harrison and colleagues, germ-free mice colonized with single commensal strains can be used for the identification of clonally expanded commensal-specific T cells using scRNA-seq of TCR pairs.^43^ The detection of clonal TCR pairs can be used to guide the development of hybridoma reconstitution screening assays and for the identification of commensal-reactive TCR heterodimers. Cloning of a single commensal-specific TCR pair into a vector enables generation of TCR-transgenic mice to track commensal-specific T cells in vivo. Generation of these transgenic models represents an important tool for understanding how homeostatic encounters with commensal microbes promote the induction of commensal-specific resident T cells and to what extent the function of these cells modulates different phases of intestinal inflammation or inflammation-associated cancer.

## HETEROGENEITY OF NONHEMATOPOIETIC INTESTINAL CELLS AS REVEALED BY SCRNA-SEQ

The first study using scRNA-seq of intestinal cells was performed by Grun and colleagues and demonstrated the potential of this technology to dissect the heterogeneity of rare intestinal cell types.^44^ The study was performed using mouse intestinal organoids, which contain all the major cell types of the intestinal epithelium, and was analyzed with a modified version of the cell expression by linear amplification and sequencing method (CEL-seq). However, for the identification of rare cell types from more complex single-cell populations, the RaceID algorithm was developed.^45, 46^ This led to the identification of *Reg4* as a novel marker for hormone-producing intestinal cells, known as enteroendocrine cells.^47^ First, Reg4^+^ cells were selectively isolated, followed by use of RaceID and specific filtering criteria, resulting in the identification of novel enteroendocrine subtypes. The same method was also used in this study to characterize the intestinal stem cell population, marked by the expression of *LGR5*.^48^ RaceID classified these cells as a homogenous population, mixed with a rare population of enteroendocrine cells and Paneth cells, the latter expressing the highest levels of LGR5.

The heterogeneity of enteroendocrine cells was further explored by Yan and colleagues (2017) using scRNA-seq.^49^ Among the intestinal stem cells, Bmi1^+^ cells were found to be the most enriched for enteroendocrine markers, including transcription factor Prox1, a transcription factor previously associated with the progression of colonic cancer.^44, 50^ Prox1^+^ intestinal epithelial cells were isolated and used for scRNA-seq using a droplet-based system. Computational analysis revealed 2 subpopulations, a mature enteroendocrine-like cell subset and a subset characterized by low levels of enteroendocrine markers but coexpressing the tuft cell markers, *LGR5* and *Ascl2*, resembling secretory progenitors. These findings suggested that the enteroendocrine lineage comprises intestinal stem cells with extensive plasticity that contribute to both homeostasis and tissue regeneration.

The first extensive scRNA-seq profile of individual intestinal epithelial cells isolated from the small intestine of mice was performed by Haber and colleagues.^51^ EpCAM^+^ epithelial cells were isolated from the small intestine of C57BL/6 wild-type and Lgr5-GFP knock-in mice followed by droplet-based scRNA-seq. Unsupervised analysis identified 15 clusters that were associated with distinct cell states or cell types such as enteroendocrine, Paneth, goblet, stem, and tuft cells. Novel intestinal epithelial cell types were identified, such as 2 subtypes of tuft cells residing in the same location, one expressing neuron-related markers, the other expressing Th2-type cytokine Tslp and bearing the immune marker CD45 found for the first time in nonimmune cells. Importantly, scRNA-seq also provided novel functional insight, revealing that the remodeling processes of epithelial cells can be pathogen-specific. For example, *Salmonella* infection can induce accumulation of absorptive enterocytes and Paneth cells, whereas *Helminth* infection leads to accumulation of secretory epithelial cells. Whether these processes are the result of a direct interaction of these cells with pathogens or more complex cross-talk with resident immune cells remains to be characterized.

ScRNA-seq has also been employed to interrogate subsets of intestinal stem cells with enriched expression of MHC-II molecules that present antigen to CD4^+^ T cells, uncovering their effects on intestinal epithelial cell remodeling. Regulatory T cells are important for maintaining intestinal stem cells, and their ablation or dysregulation causes intestinal stem cell differentiation, an effect that can be further enhanced by Th1, Th2, and Th17 cells.^52^ ScRNA-seq of intestinal organoids cultured with polarized Th subsets showed how Th17 cells reduce stem cell renewal and Th1 and Th2 suppress stem cell renewal and promote differentiation of Paneth cells and tuft cells, respectively. These findings are relevant for understanding how different pathogens responsible for priming type-specific T helper responses with their associated cytokines profiles can modulate stem cell differentiation and remodeling.

## REMODELING OF EPITHELIAL AND MESENCHYMAL CELLS IN INTESTINAL INFLAMMATION

Novel mechanisms for intestinal epithelial regeneration have also been defined with scRNA-seq, through profiling of the regenerating mouse intestine. This led to identification of a previously unknown damage-induced quiescent cell type, termed a revival stem cell (revSC).^53^ This subpopulation was identified by performing scRNA-seq on the small intestine epithelium of mice after exposure to 12 Gy of whole-body irradiation. Though revSCs seemed to be extremely rare in homeostatic conditions, after intestinal injury these cells were found to reconstitute the LGR5^+^ crypt-base columnar cells, leading to regeneration of a functional intestinal mucosa barrier. The identification of revSC cells was further validated using other methods to induce intestinal injury and acute inflammation, such as treatment with dextran-sodium sulfate (DSS) or targeted ablation of LGR5^+^ crypt-base columnar cells. These cells were characterized by expression of genes involved in inflammatory responses, DNA damage, or cell survival. In particular, high expression of clusterin was noted, which encodes a stress response gene important for cell survival and distinguishes these cells from LGR5^+^ cells.^54^ Thus, scRNA-seq provided a detailed map of tissue remodeling in the damaged intestine and defined novel mechanisms that reconstitute LGR5^+^ crypt-base columnar cells to repair the intestine.

ScRNA-seq has been recently employed by our group to describe the extent of human colonic cell heterogeneity and define the presence of a crypt differentiation hierarchy in both healthy individuals and patients with ulcerative colitis (UC).^6^ Epithelial cells were isolated from colonic biopsies of healthy individuals or patients with UC collected from clinically inflamed and noninflamed mucosa. Enteroendocrine subpopulations identified were divided into L cells, enterochromaffin cells, and precursor-like cells. Five clusters of undifferentiated cells were identified, including stem cells, early transit-amplifying cells, transit-amplifying-like cells, and secretory and absorptive lineage precursor cells. A novel population of absorptive colonocytes were characterized by the expression of the calcium-sensitive chloride channel BEST4,^55^ cathepsin E, and OTOP2 markers,^56^ designated for this reason as BEST4/OTOP2 cells. Characterization of this population by scRNA-seq was also confirmed by a more recent study performed by Smillie and colleagues.^8^ Functionally, the BEST4/OTOP2 population conducts protons into the cell cytosol in response to a lowering of extracellular pH and may maintain luminal homeostasis through selective expression of uroguanylin, required for guanylate cyclase 2C activation.^57^ This population has been found to be depleted in UC, suggesting the guanylate cyclase 2C activation pathway is also dysregulated. This provides a potential rationale for the use of uroguanylin mimetics in further experimental studies. Two additional subpopulations were identified in inflammation: inflammation-associated goblet cells and intraepithelial immune cells. The greatest changes in gene expression between health and UC were observed in colonocytes and crypt-top colonocytes followed by goblet cells, stem cells, BEST4/OTOP2 cells, and enteroendocrine cells with an overall upregulation of inflammatory markers in UC, including IFNγ signaling, pro-inflammatory cytokines, and antigen presentation. Finally, this work defined a novel functional role for the colonic goblet cell–secreted antibacterial protein WFDC2 in the mucosal barrier. The protein WFDC2 was found to be important in mucus layer formation, with WFDC2 expression reduced in UC. This may at least partly explain the breakdown of the epithelial barrier observed in UC, allowing bacterial invasion and interaction with the epithelial and immune cells of the intestinal mucosa.

Microfold (M) cells are specialized in the recognition and phagocytosis of pathogenic or commensal microbiota and, at steady-state, represent around 10% of follicle associated epithelia.^58^ ScRNA-seq revealed the expansion of these cells in UC, with a transcriptomic signature characterized by strong upregulation of chemokine genes. This suggests an important role for M cells in the recruitment of immune cells to the inflamed colon in UC. Further, M cells were found to have the highest expression of risk genes implicated by genome-wide studies for IBD, suggesting these cells may have other important roles in UC, including transcytosis and antigen delivery.^8^

Mesenchymal cells represent a heterogenous population of nonepithelial and nonhematopoietic cells in the intestinal lamina propria.^59^ These cells play important roles in immune regulation and maintenance of the epithelial barrier. ScRNA-seq has been employed to characterize colonic mesenchymal subsets and define their functional contribution in both experimental colitis and patients with IBD.^7^ Although major intestinal tissue stromal cell subsets were previously classified as fibroblasts, alpha smooth muscle actin-expressing myofibroblasts and perivascular pericytes,^60, 61^ 4 subsets of fibroblasts and a niche population expressing *SOX6*, *F3*, and *WNT* genes, were identified using scRNA-seq. The latter is located in close proximity to epithelial cells and was found to be important for their renewal. This niche population was both less frequent and dysregulated during colitis, and it was hypothesized this may contribute to epithelial barrier breakdown. In addition, the crypt niche mesenchymal cells were found to be largely equivalent between mouse and human, in contrast to other mesenchymal subpopulations that showed a lack of homology. In IBD, the emergence of a novel stromal population was identified, enriched for pro-inflammatory fibroblastic reticular cell (FRC)-associated genes, lymphocytes trafficking cytokines and chemokines genes, and T-cell costimulatory TNF family ligands (ie, *TNFSF14/LIGHT*). Interleukin (IL)-33 was also enriched in this population, a cytokine strongly associated with UC that may have both pathogenic and protective functions during different phases of intestinal inflammation.^62–65^ This expanded population of cells likely matches a subset of inflammation-associated fibroblasts (IAFs) that was also found to be expanded in UC and was described more recently again using scRNA-seq. Inflammation-associated fibroblasts comprise WNT2B^+^ and WNT5B^+^ subsets, with both enriched in UC and expressing inflammation and fibrosis-associated genes including *IL-11*, *IL-24*, and *IL-13RA2*. Inflammation-associated fibroblasts were also found to be enriched in expression of *OSMR*, the receptor for the cytokine Oncostatin M (OSM).^66^ Importantly, high expression of OSM has been demonstrated to predict anti-TNF response in patients with IBD.^67^ However, the specific cell source of both the cytokine and its receptor was unknown until scRNA-seq revealed that *OSM* is mostly enriched in inflammatory monocytes, whereas *OSMR* is mostly enriched in IAFs; both of these subsets are expanded in UC.^8^ This may also explain how remodeling of intestinal subpopulations in IBD may correlate with failure of anti-TNF therapy.

Thus, scRNA-seq has enabled definition of the remodeling of both epithelial and mesenchymal cell populations during intestinal inflammation and delineated their divergence between different species. This can be used to inform the development of novel therapies, including improvement of the design of experimental models.

## TRANSCRIPTIONAL SIGNATURES OF INTESTINAL IMMUNE CELLS IN HEALTH AND INFLAMMATION REVEALED BY SCRNA-SEQ

It is clear that each individual component of the resident intestinal immune populations has a potentially important role in tissue homoeostasis, protection against invading pathogens, and regeneration of the intestinal barrier.^68, 69^ However, the molecular characteristics of these resident immune cells, and definition of their roles in both health and disease are poorly mapped.

### Macrophages

Tissue-resident macrophages originate from circulating monocytes that enter the gut and mature in the lamina propria. Intestinal resident macrophages acquire a regulatory program important for limiting the production of inflammatory cytokines and maintain intestinal barrier function through production of antimicrobial factors and clearance of invading bacteria.^70^ The use of scRNA-seq has recently revealed the identity of a subpopulation of tissue-resident macrophages with transcriptional signatures so different from inflammatory macrophages that it was observed in both healthy intestine or lesions of CD patients.^71^ ScRNA-seq was also employed to analyze control or human macrophages exposed to butyrate, a short-chain fatty acid derived from bacterial fermentation of dietary fibers in the intestinal lumen.^72^ Computational analysis revealed a clear transcriptomic signature of butyrate macrophages characterized by the expression of antimicrobial genes, particularly in subsets of more differentiated macrophages. This helped to determine how, during the process of monocyte to macrophage differentiation, butyrate drives a protective program involving LC3-associated host defense that may have important implications for clearance of invading bacteria.^73^ Boosting this antimicrobial function using butyrate may therefore represent a potential tool to prevent the onset of intestinal inflammation.

### Innate lymphoid cells

Innate lymphoid cells (ILCs) play important roles in intestinal homeostasis and contribute to protective responses against invading pathogens. Dysregulation of intestinal ILC function has been associated with both the pathogenesis of IBD and colitis-associated cancer.^74–77^ Different ILC subsets are enriched in specific intestinal locations, with ILC1 enriched in the upper gastrointestinal tract, ILC2 present throughout the entire intestine, and ILC3 mostly enriched in the colon.^78^ ScRNA-seq has been used to generate the first transcriptomic atlas of the innate lymphoid cells residing in mouse small intestine lamina propria.^79^ Massively parallel scRNA-seq (MARS-seq) was used to obtain unbiased transcriptomic signatures of CD127^+^ cell populations isolated from the lamina propria.^80^ This approach led to the identification of 15 ILC subgroups, including 4 distinct ILC1, 4 ILC2, 5 ILC3, and 2 clusters that did not have a clear phenotypic association with any conventional ILC subgroup. In addition to a deep characterization of the ILC landscape, the study also demonstrated that the responsiveness of ILCs to the microbiome is highly heterogeneous. Indeed, homeostatic exposure to commensal colonization may suppress the acquisition of a transcriptional program associated with ILC3 profiles,^81^ as confirmed by the fact that germ-free mice or antibiotic-treated mice acquire genomic elements typically associated with ILC3 profiles.^79^ ScRNA-seq has been more recently employed to characterize the transcriptional signatures of ILC2 cells from the mouse small intestine at steady state and after induction of type 2 inflammation. This study demonstrated how inflammation-mediated expression of the alpha-calcitonin gene-related peptide (α-CGRP) in ILC2 modulates their responses at the intestinal level.

### Lymphocytes

When considering tolerogenic immune cells, regulatory T cells (Tregs) represent a specialized subset of CD4^+^ T cells that play an essential role in intestinal homeostasis.^82, 83^ Tregs express high levels of transcription factor Foxp3, considered important for their immunoregulatory function.^84–85^ In mice, this population is enriched within the CD45RB^low^ and CD25^+^ subsets and inhibits colitis, as demonstrated by experimental colitis induced by T-cell adoptive transfers.^86^ Trafficking of Tregs to nonlymphoid tissues occurs in response to varied stimuli including commensal bacteria and dietary-derived antigens. Trafficking of both these cells and other types of lymphocytes requires a specific transcriptional program that allows the acquisition of tissue-specific cellular adaptation that differs from their counterparts in lymphoid tissues.^87, 88^ Using scRNA-seq in mice, Miragaia and colleagues identified transcriptionally distinct subsets of Tregs in nonlymphoid barrier tissues, such as colon and skin, and showed their adaptation to these sites during their transition from lymphoid tissues.^89^ Interestingly, adaptation of Treg cells to skin and colon relies on a similar transcriptional program. In the colon, 3 subsets of Tregs were found and named as nonlymphoid tissue, suppressive, and lymphoid-tissue like. The most highly expressed genes involved in the adaptation program to nonlymphoid tissues involve TNF receptors (ie, *TNFRSF9*), which highlights the importance of the TNFRSF-NF-κB pathway for the identity of these cells in barrier tissues. In addition, scRNA-seq was employed in the same study to compare murine and human infiltrating Treg cells in colon and skin. In the colon, 17 of 74 human Treg cell markers overlapped with their respective murine markers. The majority of these markers were related to the TNFRSF-NF-κB pathway, confirming the importance of this conserved program in both mice and humans. Interestingly, a separate scRNA-seq study revealed that Tregs markedly expand in inflamed colonic tissue of patients with UC and become major sources of TNF.^8^ Whether TregTNF^+^ cells contribute to disease or determine response to anti-TNF therapy is yet to be determined. In this context, the utility of scRNA-seq has been demonstrated in predicting response to anti-TNF therapy in pediatric Crohn’s disease. This study identified an inflammatory cellular module in inflamed tissues consisting of IgG plasma cells, inflammatory macrophages, activated T cells, and stromal cells named the GIMATS module. The presence of GIMATS in a subset of patients predicted failure to achieve durable corticosteroid -ree remission with anti-TNF therapy.^71^

Although CD4^+^ T cell responses are conventionally thought to be associated with pathogenesis of IBD, transcriptional profiling of circulating T cells isolated from patients with IBD has previously shown a CD8^+^ T cell signature associated with more severe disease.^90^ Further, adoptive transfer of naïve CD8^+^ T cells to immunodeficient mice results in colitis in vivo.^91^ It is clear that innate immune pathways affect the nature of CD8^+^ T cell response. For example, NOD2 (the most strongly associated Crohn’s disease susceptibility gene) can affect MHC class I responses through cross-presentation pathways, and in the presence of Crohn’s-associated NOD2 polymorphisms, these responses are dysregulated.^92^ ScRNA-seq has recently revealed the expansion of a CD8^+^ subpopulation expressing IL-17 in UC.^8^ However, the specific role of these subpopulations in the modulation of gut inflammation remains to be elucidated.

A further important class of resident immune cells are resident memory T cells, which migrate into nonlymphoid tissues, particularly the intestine, and do not recirculate.^93^ These cells typically originate from CD8^+^ effector T cells, which are particularly abundant in the intestine, where they provide rapid and highly efficient responses independently of circulating T cells.^94^ Human resident CD8^+^ T cells, expressing *ITGAE* (CD103) when activated ex vivo, are potent cytokine-producing cells when compared with CD103^-^ CD8^+^ T cells, which are a subset more similar to their circulating counterpart.^41^ Intraepithelial lymphocytes (IELs) represent a further important resident immune cell class.^95^ ScRNA-seq analysis combined with immune phenotyping (TCR profiling) has been recently used to characterize cellular heterogeneity of a cohort of pediatric patients with colitis and IBD. This study showed the preferential clonal expansion of two subsets of CD8^+^ T cells expressing *ITGAE* and in CD8^+^ T cells expressing *GZMK*. Immune phenotyping of IELs showed that the proportion of resident CD8^+^ T cells expressing *ENTPD1* (CD39) and γδT cells is significantly decreased in the colonic mucosa of children with colitis and IBD. Deficiency of CD39 in CD8^+^ T cell may be due to defective cAMP response in pediatric patients with colitis and IBD, and it may be responsible for exacerbating inflammation via platelet aggregation and release of 5-hydroxytryptamine. In line with this, the antiplatelet drug dipyridamole increased CD39 in CD8^+^ T cells and alleviates clinical symptoms for children with colitis.

In summary, the development of scRNA-seq studies is leading not only to redefinition of the molecular characteristics of intestinal resident immune cells in health and IBD but also to identification of novel biomarkers as an opportunity to develop novel drug targets or to use them as predictors of response to current therapies.

## SINGLE-CELL EPIGENETICS

It is increasingly clear that epigenetics, defined as modifications in gene expression that are controlled by heritable but potentially reversible changes in DNA methylation or chromatin structure without altering the DNA sequence, are important in the pathogenesis of IBD.^96–98^ Further, epigenetic markers have emerged as a powerful predictor of disease course and the need for treatment escalation in IBD.^99^ Importantly, epigenetic modifications can be both tissue- and cell-type specific, and thus traditional epigenetic analysis using bulk measurements of heterogenous cell populations may easily miss functionally critical epigenetic marks.^96, 100^ This is of particular relevance when considering the epigenome of rare cell populations.

Recent studies have reported remarkable advances in single-cell epigenetic technology, providing the opportunity to analyze single-cell DNA methylation and chromatin structure in tens of thousands of cells.^101–104^ However, markedly different biochemical approaches are required to interrogate each form of epigenetic modification, as they range from covalent DNA modifications to chromatin accessibility and conformation.^105^ DNA methylation can be assessed using bisulphite (BS) conversion of cytosine residues, which is the most common methylated base. Although early single-cell BS-seq was limited by poor read coverage, recent modifications have allowed up to 70% read alignment.^102^ Further, BS-seq and RNA-seq can now be combined in the same single cell, allowing simultaneous interrogation of associations between epigenetic and transcriptional variation^106^ (Fig. 2). Increased chromatin accessibility is a key feature of cell type specific cis-regulatory elements of transcription. Assay for transposase-accessible chromatin followed by sequencing (ATAC-seq) and DNase-seq both provide assessment of genome-wide chromatin accessibility and can be combined with microfluidics to separate single-cell nuclei and perform reactions in individual cells^107, 108^ (Fig. 2). However, these approaches do not capture repressive chromatin states that may be of equal functional importance. Modulation of chromatin structure through histone modification is a further key mechanism of epigenetic regulation.^100^ This can now also be studied on a single-cell level using chromatin-immunoprecipitation followed by sequencing (ChIP-seq). A recent study in breast cancer cells demonstrated the ability of single-cell ChIP-seq to identify rare heterogenous chromatin states associated with treatment resistance that would be missed with traditional bulk approaches^109^ (Fig. 2). DNA adenine methyltransferase identification (DamID) is a further technique for studying protein-DNA interactions without the need for antibodies or immunoprecipitation and has been used to map nuclear lamina interactions in single human cells from a human myeloid leukemia cell line.^110^ Finally, single-cell combinatorial indexed Hi-C (sciHi-C) allows study of chromosome conformation and interaction, a further important epigenetic regulator that previously could only be studied using microscopy. This method uses proximity ligation to profile chromosome conformation, with combinatorial cellular indexing recently applied to analyze thousands of cells simultaneously from human cell lines^111^ (Fig. 2).

In summary, although single-cell epigenetic technologies have not yet been utilized in the study of IBD, it is a rapidly evolving field that shows rich promise. Looking to the future, the hope is that experimental workflows that integrate DNA methylation, chromatin accessibility, histone modification, and gene expression will be developed, allowing comprehensive assessment of the epigenome and interacting transcriptome at single-cell level.

## CLINICAL APPLICATIONS OF SINGLE-CELL STUDIES—FROM THE BENCH TO THE BEDSIDE

Inflammatory bowel disease follows a relapsing and remitting pattern, with unpredictable disease course and strikingly heterogeneous clinical outcomes, even when Crohn’s disease and ulcerative colitis are considered separately. Even though the advent of biological therapy has revolutionized care, only 30%–40% of patients show a sustained response to any class of therapy.^112^ Current treatment algorithms rely on a “trial and error” approach, and thus patients may receive ineffective and costly therapy for many months with associated risks. Conversely, patients who may otherwise follow a relatively benign disease course are at risk of overtreatment if early aggressive “top-down” therapy, where potent immunosuppressants are introduced early, is selected. There is a pressing need for technologies that allow us to predict disease course and response to therapy, ensuring that the right patient receives the right intervention at the right time—a central aim of personalized medicine.^113, 114^

**FIGURE 3.**
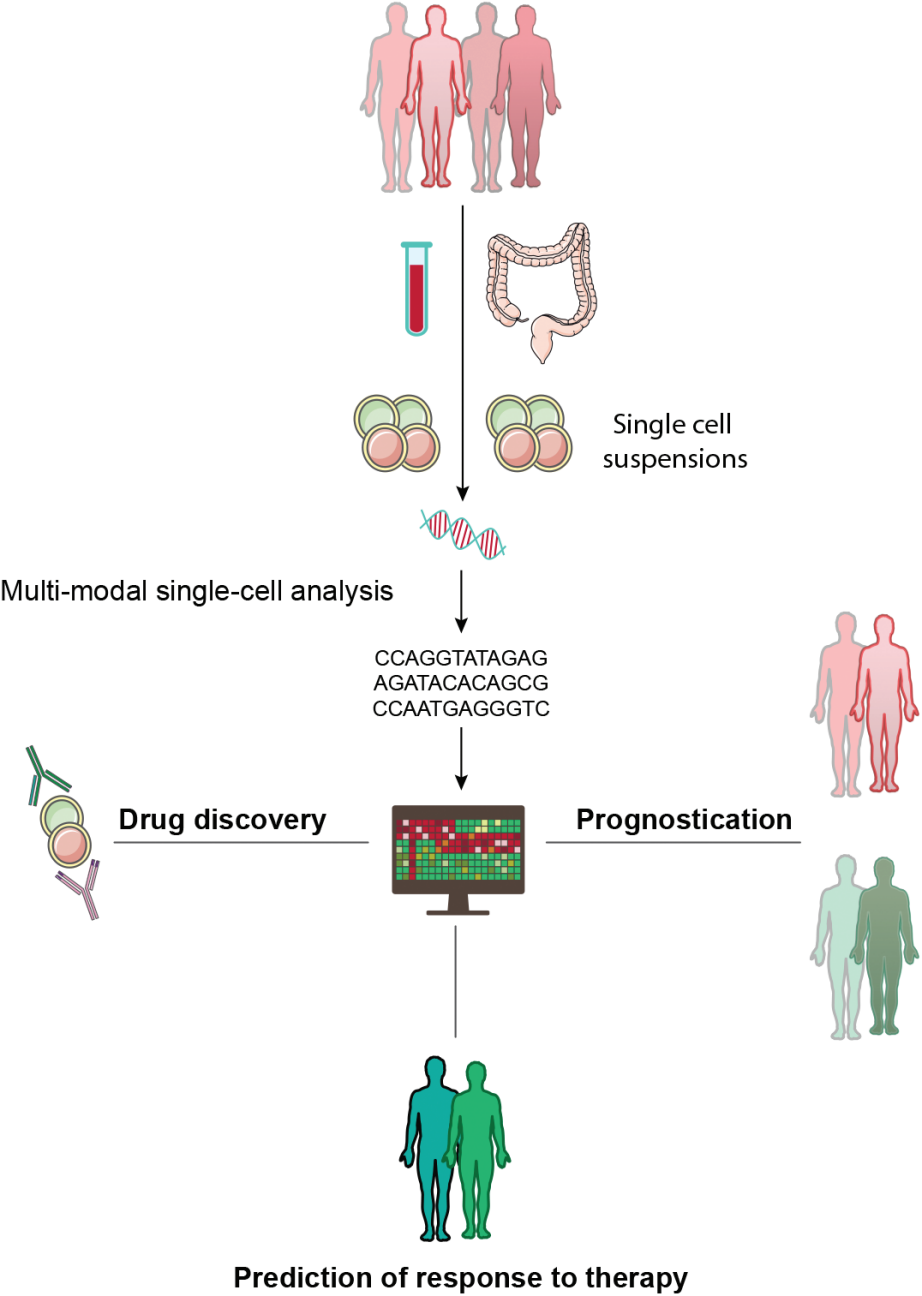
Personalization of care in IBD using single-cell technology. Multimodal single-cell analyses allow precise interrogation of individual cell populations derived from peripheral blood and intestinal samples in patients with IBD. These data will drive prognostication of disease course, prediction of response to therapy, and novel drug discovery.

The strength of single-cell technology lies in its ability to accurately characterize the molecular and functional heterogeneity of individual cell populations, and it is now beginning to shine light on the considerable pathophysiological darkness of IBD, opening novel opportunities for drug targets^6–8^,^115^ (Fig. 3). Further, poorly defined pathophysiological heterogeneity between patients is hypothesized to be a major limiting factor in the success of drug trials, leading to the premature discarding of therapeutic avenues that might have proved effective in carefully selected subgroups of patients.^7^ The further utility of single-cell technology in predicting response to therapy has recently been demonstrated in pediatric Crohn’s disease. Martin et al identified an inflammatory cellular module in affected ileum, consisting of IgG plasma cells, mononuclear phagocytes, activated T cells, and stromal cells that predicted failure to achieve durable corticosteroid-free remission with anti-TNF therapy.^71^ In the years to come, single-cell technology will seek to harness a multi-omics approach, integrating genetic, epigenetic, transcriptomic, proteomic, and microbial classification of disease and predictors of outcome and response to therapy, which may finally usher in the long awaited era of precision medicine in IBD (Fig. 3).

However, significant challenges must be overcome before single-cell technologies can be routinely utilized in clinical practice. The first relates to expense, although costs will undoubtedly fall in the years ahead, and must be weighed against the current substantial direct health care costs of IBD care, estimated to be $22,987 per patient per year in the United States.^116^ The second relates to the availability and scalability of the workflow system, including the complexity of data analysis, but the rapid progression in technology suggests that compact single-cell work stations utilizing largely automated analysis is a realistic target, with vast data sets reduced to simple immune monitoring panels.^117^ The third obstacle is the complex area of intellectual property, but this is not a challenge unique to single-cell technology, and collaborations between clinicians, scientists, and industry will continue to drive progress for our patients.

## Notes

Author Contribution: Authors contributed equally to the manuscript. All authors edited and approved the final version.
